# Inheritance metrics feats in unsupervised learning to classify unlabeled datasets and clusters in fault prediction

**DOI:** 10.7717/peerj-cs.722

**Published:** 2021-10-29

**Authors:** Syed Rashid Aziz, Tamim Ahmed Khan, Aamer Nadeem

**Affiliations:** 1 Department of Software Engineering, Bahria University, Islamabad, Pakistan; 2 Department of Software Engineering, Capital University of Science & Technology, Islamabad, Pakistan

**Keywords:** Software metrics, Software inheritance metrics, Machine learning, Software fault prediction, Supervise learning

## Abstract

Fault prediction is a necessity to deliver high-quality software. The absence of training data and mechanism to labeling a cluster faulty or fault-free is a topic of concern in software fault prediction (SFP). Inheritance is an important feature of object-oriented development, and its metrics measure the complexity, depth, and breadth of software. In this paper, we aim to experimentally validate how much inheritance metrics are helpful to classify unlabeled data sets besides conceiving a novel mechanism to label a cluster as faulty or fault-free. We have collected ten public data sets that have inheritance and C&K metrics. Then, these base datasets are further split into two datasets labeled as C&K with inheritance and the C&K dataset for evaluation. K-means clustering is applied, Euclidean formula to compute distances and then label clusters through the average mechanism. Finally, TPR, Recall, Precision, F1 measures, and ROC are computed to measure performance which showed an adequate impact of inheritance metrics in SFP specifically classifying unlabeled datasets and correct classification of instances. The experiment also reveals that the average mechanism is suitable to label clusters in SFP. The quality assurance practitioners can benefit from the utilization of metrics associated with inheritance for labeling datasets and clusters.

## Introduction

Computers have proven to be ubiquitous in everyday life, and software development has also become a key economic activity, aimed at providing reliable and high-quality software with the least possible errors or defects (Chiu, 2011). Standish Group reported that in 2009, only 32% of projects of software were completed within cost and time, about 44% projects are in conflicting state and 24% projects have been terminated (Dominguez, 2009). An error can be described as a state in the code, blocking the software to perform according to specifications. Generally, faults are also identified as failures or errors and these terms are used interchangeably (Bell, Ostrand & Weyuker, 2013). Since the faulty modules of software can lead to software crashes, rise costs of development, and maintenance with the dissatisfaction of the customer. So useful models of fault prediction can support the development teams to concentrate on quality assurance activities to focus on the fault-prone classes, thereby perfecting the quality of software by managing resources competently (Koru & Liu, 2005).

Software fault prediction (SFP) is generally considered to be a binary classification activity in which the classes denoted by code attributes or metrics are grouped into two classes, for example not prone to a fault (nfp) and prone to a fault (fp) employing a classification model originated on the data of earlier projects of software development (Schneidewind, 1992). Wide-ranging work has been performed to deliberate the numerous classifiers applied in the field of SFP, that may be separated into two categories, namely statistical approaches comprising discriminant analysis (Briand, Brasili & Hetmanski, 1993; Schneidewind, 2001), logistic regression (Denaro, Pezzè & Morasca, 2003; Khoshgoftaar, Seliya & Gao, 2005) and Bayesian networks (Menzies et al., 2004; Turhan & Bener, 2009), and as models of machine learning comprising methods such as based on rules/tree structure (Koru & Liu, 2005; Arisholm, Briand & Johannessen, 2010), SVM (Xing, Guo & Lyu, 2005), neural networks (Kanmani et al., 2004; Zheng, 2010), fuzzy logic methods (Zhong, Khoshgoftaar & Seliya, 2004), analog approaches (Ganesan, Khoshgoftaar & Allen, 2000) and methods of set (Catal & Diri, 2009a). According to the Catal and Diri literature review, algorithms of machine learning are the utmost prevalent approach to predicting faults (Catal & Diri, 2009b).

The software fault prediction model generally needs a dataset of an earlier software project for the training of the model (Punitha & Chitra, 2013; Petersen, 2011). There are generally two approaches depending on dataset availability for model development. Supervised learning is the first approach, wherein the model for predicting software defects is built through the training data set and assessed by the test data set. The unsupervised is the second approach, in which the model for predicting faults in software is built employing the existing test dataset deprived of a training dataset.

Presently there are two forms of fault prediction models in the supervised approach, fault predicting within a project, and cross-project fault prediction (Zhang et al., 2016). There is an obstacle to use the first model on a newly created software project because it has no previous training data sets (Zhang et al., 2016; Nam et al., 2017). As a result, a fault prediction cross-project model is utilized to solve this problem, in which a training dataset is retrieved from some other projects of software to construct a model (Nam et al., 2017; Singh, Verma & Vyas, 2014; Ryu & Baik, 2016). Despite this, transferring analogous training data records by some other project is not straightforward for the reason of diversity between the source project and target projects (Zhang et al., 2016; Cheng et al., 2016; Yeh, Huang & Wang, 2014). The approach of unsupervised learning suggests a substitute way out to the problem of accessibility of training datasets (Fu & Menzies, 2017; Yang & Qian, 2016).

Regarding the use of an unsupervised approach, the predicting model may be immediately formed to use the current software data set without the need for training data sets (Wahono, 2015). This data set comprises the onset of unlabeled classes. The said method depends on clustering to classify the unlabeled data set to divergent clusters, that are faulty and fault-free (Li et al., 2014). Every entity in both the clusters is labeled using a classifier (Zhang et al., 2016; Marjuni, Adji & Ferdiana, 2019).

In general, unsupervised learning methods use clustering methods, for example, K-means, DBSCAN, hierarchical, Cobweb, CLOPE, and OPTICS algorithms. The algorithm of clustering has classified similar instances together in a similar group. The problem at this point is the number of clusters has to be chosen heuristically which will affect the results of the predicting model. In addition to choosing a number, the researcher has to do this tedious job several times to find optimum results.

Public and private data sets have been studied in the field of SFP because some people think that private data research is an additional challenge to validate (Catal, 2011; Catal & Diri, 2009b). Public data sets come mainly from the tera-PROMISE (Shirabad & Menzies, 2005) and NASA MDP (metric data program) (Chapman, Callis & Jackson, 2004) repositories, these are freely accessible by researchers, and therefore gain significant attention (Catal, 2011; Catal & Diri, 2009b; Cukic & Ma, 2007; Menzies, Greenwald & Frank, 2006).

Software metrics are utilized to predict whether a class in software is faulty or fault-free. These metrics can be utilized in clustering algorithms, which apply numerous distance measures to decide clustering distances. Many different metrics can be used to predict faults, such as coupling, inheritance, C&K metrics suit, etc. A review of the literature reveals that the metrics suite of Chidamber & Kemerer (C&K) is extensively applied in SFP. This combination consists of six metrics, namely {noc, wmc, dit, cbo, lcom, rfc}.

During a survey on fault prediction, it is revealed that a lot of efforts are previously made by the researchers on cohesion, coupling, and other metrics in the perspective of the Object-Oriented paradigm. Most of these are utilized individually or in a group with other metrics in the experiments. The best example is the C&K metrics suite in which most of these are used in a group and benchmarked in the experimental studies. It is noticed that individual utilization and assessment of inheritance metrics are lacking in the research, so basing on this reason, there a need to perform an experiment aiming exclusively at the inheritance aspect to depict the usefulness of its metrics to predict faults (Aziz, Khan & Nadeem, 2020).

The primary objective of this paper is to experimentally prove the effectiveness of inheritance metrics to handle unlabeled datasets in SFP applying unsupervised learning and the secondary objective is to the identification of clusters as faulty or fault-free through a novel average mechanism.

The paper is divided into six sections, Section two is related work that explains clustering methods, K-means, model evaluation, C&K metrics, and inheritance metrics. Section three is a literature review that explains clustering and inheritance in software fault prediction. Section four explains the experiment aspects. Section five is addressing threats to validity, and Section six is the conclusion and future work.

## Related work

### Clustering methods

An approach where instances are grouped into a cluster is referred to as unsupervised learning. It is situated in the secondary category of data mining, whereas according to the classification, it is in the primary category of data mining. Since, classification utilizes labels of the class for training, whereas these labels do not utilize in the clustering rather attempt to find out the similarity among the features (Gan, Ma & Wu, 2007). Methods of clustering may be utilized to cluster classes with alike metrics through measuring distances or similarities. Subsequently to apply clustering methods, the average worth of all metrics contained by the cluster be able to examine besides the industrial thresholds of a metric. If the boundaries go beyond the value, the cluster could be categorized as faulty. The cluster analysis encompasses four fundamental points (Xu & Wunsch, 2005):

1. **Selection of feature.**
2. **Selection of clustering algorithm.**
3. **Cluster validation.** The clustering algorithm may produce numerous clusters, but these may not reveal the actual patterns situated in the data set. Thus, parameters of evaluation are essential to evaluate the algorithm efficiency.
4. **Results Interpretation.**

The grouping of clustering algorithms is not simple. The classification formed by Berkhin (2006) is shown in Table 1.

**Table 1:** Classification of clustering algorithms.

Methods	Algorithms
Hierarchical methods	Agglomerative algorithms
	Divisive algorithms
Partitioning methods	Relocation algorithms
	Probabilistic clustering
	K-medoids methods
	K-means methods
	Density-based algorithms
	Connectivity clustering
	Density functions clustering
Grid-based methods	
Methods using co-occurrence of categorical data	
Constraint-based clustering	
Clustering algorithms used in machine learning	Gradient descent and neural networks
	Evolutionary methods
Scalable clustering algorithms	
Algorithms for high dimensional data	Subspace clustering
	Projection techniques
	Co-clustering techniques

The above grouping may overlay, and some other scholars may generate diverse classes of these algorithms. Alternative grouping (Gan, Ma & Wu, 2007) is depicted in Table 2.

**Table 2:** Alternative classification of clustering algorithms.

Fuzzy clustering		
Hard clustering	Partitional	K-means and derivatives
		Locality-sensitive hashing
		Graph-theoretic methods
	Hierarchical	Divisive
		Agglomerative
Graph methods		
Geometric methods		

### K-means

K-means is named Lloyd’s algorithm alternatively, mainly in the computer science community. It is common for the analysis of clustering in data mining. Its algorithm classifies entities in k clusters. The basic idea is to outline k centroids one for each cluster. The finest selection is to place these as farthest away from each other as feasible. The next step is to take every instance of the specified data set and correlate it by the adjacent cluster. Once all is done the first step is finished. After this, we need to compute again k new center of each cluster. After having a new center of k, we must assign points among the same datasets and adjacent to a new center. The midpoint of k amends its location gradually till then there are no variations exist or simply the midpoint is not rotating. Mostly, K-means is faster computationally as compared to hierarchical cluster and it creates clusters which are too nearer than the hierarchical cluster, particularly if the clusters are globular. The K-means is useful in data compression, data modeling, expression analysis, and other areas. The clustering process is explained as under:

1. The first step is to set the value of K—select quantity of clusters denoted as k.
2. The next step is Initialization—To select k initial position that is utilized as starting midpoint of a cluster.
3. Next step is classification—where mark every data point to the cluster that Euclidean distance is small among midpoint of cluster and point.
4. Next step is Centroid calculation—Once all point in the data set is assigned to a cluster, there is an essential process to again compute the new k midpoint (centroids).
5. Next step is convergence criteria—The steps of (iii) and (iv) are expected to be repeated until the midpoint (centroids) are not shifting.

The pseudo code of the algorithm is shown in Algorithm 1 (Gan, Ma & Wu, 2007). During the first phase of initialization, clusters are initialized randomly through the occurrences. Subsequent to this iteration phase where the assignment of instances is computed according to distances formula and then allocated to the nearest cluster. Distance is computed in-amongst the occurrence and the midpoint of the cluster. This phase iteratively performed until midpoint is not shifting furtherer.

**Algorithm 1:** PseudoCode of K-means clustering method.

1 function K-MeanClusteringFun
**Input :** Require: D Dataset, k number of clusters, d Dimension
2 {Ci is the ith cluster}
3 {A. Phase of Initialization}
4 (C I, C II, …, Ck} = Starting partition of dimension d.
5 {B. Repetitive Phase}
6 repeat
7 dnm = interval among case n and cluster m;
8 xi = arg minim dnm;
9 Allocate case i to cluster xi;
10 Recalculate the means of cluster due to center point changed;
11 till no more variations of cluster associates arise
**Output :** Cluster results

Many distance functions are used in the literature some of them are listed as under:

1. Euclidean distance.
2. Manhattan distance.
3. City block distance.

### Model evaluation metrics

In the SFP domain, diverse measurements to gauge the performance constructed from the confusion matrix are utilized to describe and assess the results of numerous techniques (He & Garcia, 2009; Wang & Yao, 2013; Arar & Ayan, 2015; Kumudha & Venkatesan, 2016). Table 3 demonstrates a conventional confusion matrix where the fault-prone and the fault-free are measured as positive and negative, respectively. The confusion matrix consists of four sections for binary classification which are the number of positive classes truly classified (TP), the number of falsely classified positive classes (FP), the number of falsely classified negative classes (FN), and the number of truly classified negative classes (TN) (Henein, Shawky & Abd-El-Hafiz, 2018).

**Table 3:** Confusion matrix.

	Actual faults	Actual faults-free
Predicted faults	TP	FP
Predicted fault-free	FN	TN

In order to measure the proposed software fault prediction model, we consult the confusion matrix that appeared in Table 3, which is a key source for accuracy assessment in classification problems. Based on the confusion matrix, the following measures are utilized for appraisal (Alsawalqah et al., 2020):

1. Recall. Recall is the segment of applicable occurrences that are retrieved over the sum of applicable occurrences for example coverage rate. This may be described with the equation mentioned as under:
$Recall = \displaystyle{{TP} \over {TP + FN}}$
2. Precision: The Precision is a percentage of applicable occurrences amongst the retrieved occurrences. This may be described with the equation mentioned as under:
$Precision = \displaystyle{{TP} \over {TP + FP}}$
3. F1-measure. F1-measure is computed based on confusion matrix elements as mentioned above. F1-measure nearer to a means of the classifier has excellent performance prediction. This may be described with the equation mentioned as under:
$F1\text{-}measure = \displaystyle{{2*\left(\displaystyle{{TP} \over {TP + FP}}\times\displaystyle{{TP} \over {TP + FN}}\right)} \over {\left(\displaystyle{{TP} \over {TP + FP}} + \displaystyle{{TP} \over {TP + FN}}\right)}}$
4. Receiver Operating Characteristics Curve (ROC). The curve ROC demonstrates the trade-off between the false alarm rates (PF) and the probability of detection (PD). It is utilized to measure the performance of the classifier around altogether likely assessment thresholds. The range of area under the curve (AUC) is positioned within 1 and 0 (Bradley, 1997; Huang & Ling, 2005). It is equal to the probabilities of the arbitrarily selected example of the positive class that will have lesser predictable probabilities to be misclassified than an arbitrarily selected example of the negative class. A decent predictor may contain a higher value of AUC.

### Metrics suite of C&K

The Chidamber & Kemerer metrics suite initially contains six metrics computed for each class, {noc, wmc, dit, cbo, lcom, rfc} (Aivosto, 2018). Each of them is briefly described in succeeding lines:

1. **Weighted Methods Per Class (WMC).** Regardless of the lengthy name of WMC, it is the counting of methods in the class.
$WMC = number\;of\;methods\;defined\;in\;class$
  Retain WMC lower as a higher value is leading towards faultiest. One technique in which to bound the number of methods within a class to 20 or 50 approximately. An alternative technique where the utmost 10% of classes may contain more than twenty-four methods. So, this permits creating larger classes but most classes will be small.
2. **Depth of Inheritance Tree (DIT).** A class is as deep within the hierarchy contains extra variables and methods the class is likely to inherit, turn it into the further complex. Deep trees usually point toward major design complexity. On the other hand, taking a positive aspect, deeper trees encourage reusability as they inherit methods.
$DIT = max\;inheritance\;path\;from\;class\;to\;root\;class$
  A higher value of DIT indicates the increase of faults so suggested DIT is five or less. Specifically, documentation of dot NET suggests that DIT be less than or equivalent to five as overly deep classes hierarchy is complicated to be developed. A few sources suggest the range up to eight.
3. **Number of Children (NOC).** NOC equal to the number of direct subclasses inherited from a base class. inheritance is not available in Visual Basic so the value of NOC is always 0.
$NOC = number\;of\;immediate\;sub - classes\;of\;a\;class$
  NOC calculates the width of a class hierarchy and DIT gauges the depth. Usually, depth is good as compared to width because it supports the method’s reusability by the use of inheritance. DIT and NOC are strongly correlated. The levels of Inheritance can be included to enhance the depth and decrease the width. The higher value of NOC shows a small number of faults. This might be due to higher reuse, which is usually desired.
4. **Coupling between Object Classes (CBO).** The classes are coupled where methods are defined within a class and use of these methods or instance variables declared by the other class. The utilization association can be in any direction: both uses and used-by relations are taken into consideration, but just once only.
$CBO = number\;of\;classes\;to\;which\;a\;class\;is\;coupled$
  A higher value of CBO is disadvantageous. Extreme coupling within object classes is damaging to modular design and avoids reusability. A higher value of coupling reveals fault-proneness. Thorough testing is therefore required. There is a question how high is excessively high? CBO less than fourteen is very high (Basili, Briand & Melo, 1996; Chidamber & Kemerer, 1994; Marinescu, 1998).
5. **Response for a Class (RFC).** Response for a class is a methods group that may be run in reply to receiving a message by a class object. RFC is the value of methods in the set.
$RFC = M + R(First - stepmeasure)$
  where M = # of methods defined within a class and R = # of outside methods explicitly called by a class methods. As RFC precisely contains methods contacted via the remote class, so that it is a measurement of possible interaction among the methods of both classes. A larger value of RFC possibly has more faults. All classes which have a higher value of RFC are difficult to understand and more complex. Therefore, debugging and testing of classes are difficult.
6. **Lack of Cohesion of Methods (LCOM).** LOCOM or LCOM is a sixth metric in the suite of C&K metrics which is expressed as the lack of cohesion of methods. The computed process is described as under:
$LCOM1 = P - Q,if\ P \gt QLCOM = 0\;otherwise$
  Choose all sets of class methods, If these access disconnect groups of instance variables, add one in P. If these share at minimum one variable access, add one to Q. LCOM = 0 shows a cohesive class. LCOM > 0 shows the need a class or class might divide into two or more classes, as its variables are suitable in disconnect groups. A class having a higher value of LCOM be discovered to be faulty.

### Inheritance metrics

1. **Measure of Functional Abstraction (MFA).** The MFA is a percentage of the number of inherited class methods to the number of methods accessible by class member methods (Chawla, 2013). Further explained as the average total classes in the design of the percentage of the number of methods inherited by a class to the sum of methods available to that class, for example, methods inherited and defined.
2. **Inheritance Coupling (IC).** The IC metric offers the amount of superclass with whom a child class is coupled. Usually, a class is coupled with the superclass in a case where at least one of his inherited methods functionally dependent on newly or re-defined class methods. Generally, a class is coupled with a superclass in the case where one clause in the following is fulfilled:
  - At least one method which is inherited utilizes a data member or a variable that is declared within a redefined or new method.
  - At least one method which is inherited communicates with a re-defined method.
  - At least one method which is inherited is contacted by a re-defined method and utilizes a variable that is declared within the re-defined method.

## Literature review

This section describes the research arena where clustering is being used in the background of software fault prediction is addressed. Additionally, enlisted the research papers exclusively focusing the inheritance aspect on fault prediction.

### Clustering

The use of unsupervised software fault prediction has been experimented with in the followings studies where numerous methods are utilized in the literature. Zhong, Khoshgoftaar & Seliya (2004) measure the algorithms natural-gas with the K-means algorithm to assess the performance of a cluster where the natural gas algorithm outperformed based on mean square error. Despite this, the technique needs a software specialist to decide about the software that is appropriate to categorize in the class of faulty or fault-free. Catal, Sevim & Diri (2009) employed clustering algorithm X-Means to find faulty or fault-free clusters applying certain software metrics as the splitting threshold. These metrics include cyclomatic complexity, operand, operator, and lines of code. The object of software is forecasted faulty in the case where the metric values are larger as compared to the threshold value, and conversely.

Bishnu & Bhattacherjee (2011) suggested the K-means algorithm based on quadtree and relate it to certain algorithms of clustering. His suggested algorithm’s error rates are rationally equivalent to Linear Discriminant Analysis, Naive Bayes, and K-means.

Abaei, Rezaei & Selamat (2013) put forward an algorithm self-organizing map for the clustering of software faults. In order to manage the label of the classes, they make use of the same threshold metrics which were utilized in the algorithm of X-Means clustering, recommended by Catal, Sevim & Diri (2009). The findings of their experiment depicted that the algorithm outclassed the algorithms of Catal, Sevim & Diri (2009) and Bishnu & Bhattacherjee (2011).

Nam & Kim (2015) suggested a threshold for clustering based on median-based partitioning. Every object having high values compared to the median is positioned in one cluster, on the other hand, all those objects are positioned in the second cluster. The elements in the upper half of the cluster are categorized as faulty, whereas the elements in the lower half of the cluster are grouped as fault-free. The suggested technique contains healthier results as compared to J48, Bayesian Network, Logistic Regression, Logistic Model Tree, Naive Bayes, Random Forest, and Support Vector Machine. Zhang et al. (2016) employed the spectral graph to build an unsupervised built on spectral classifier algorithm. The group of software objects with its connectivity depicts the edges and nodes of the spectral graph, respectively. They utilized the threshold of value zero to get the forecast faulty and fault-free clusters. In order to label entire cluster elements, they utilized the exploratory sums of row criterion. The findings of their experiment depicted that the classifier spectral performed much better as compared to logistic regression, random forest, logistic model tree, and Naive Bayes algorithms. Their advised technique is enhanced by Marjuni, Adji & Ferdiana (2019) to confirm the usage of spectral classifier needs, especially in the non-negative Laplacian graph matrix. They employed the absolute adjacency matrix to build the signed Laplacian graph matrix. The experimentation illustrations the usage of signed Laplacian on spectral classifier may expand both classification and cluster density.

Seliya & Khoshgoftaar (2007) suggested a constraint-based semi-supervised clustering scheme that utilizes clustering algorithm K-means as the fundamental algorithm aimed to handle this issue. The findings suggested that the employment of said approach superior as compared to their earlier prediction approach based on unsupervised learning. Nevertheless, the identification of a number for a cluster is until now a serious problem in this model and they have used an expert’s field knowledge in their approach to repetitively tag clusters as faulty or fault-free. Consequently, this model is also reliant on the competence of the expert.

### Inheritance

Inheritance feature is infrequently focused in the research exclusively. In this regards important conclusions drawn in the research arena are explained in the followings lines.

In a study, literature is reviewed to find out the effectiveness of inheritance metrics in the fault prediction domain. The conclusions of the study indicate that about fifty-five inheritance metrics are formed up till now by the scholars where a couple of inheritance metrics are being utilized for fault prediction. Also, 79 public data sets were found, which encompass only ten inheritance metrics with many combinations. The use of Method-level metrics is sixty percent, and the same percentage is for the usage of private data sets. It is also identified that the utilization of machine learning approaches is growing, which used several performance measures. This study will help researchers to investigate the previous studies based on software metrics, their methods, various datasets, metrics of performance evaluation, and experiment results perspectives in an easy, and effective manner specifically consideration on the Inheritance viewpoint.

Similarly, inheritance metrics viability in software fault prediction is validated through the experiment. A total of 40 datasets having inheritance metrics are collected. After preprocessing, the selected data sets have been divided into all possible combinations of inheritance metrics followed by merging similar metrics. Resultantly, 67 data sets have been formed containing only inheritance metrics that have nominal binary class labels. SVM is used for model building and validation. Results based on error rate entropy advocate the viability of inheritance metrics in software fault prediction. Furthermore, {ic}, {noc}, {dit} are helpful in the reduction of error entropy rate overall 67 feature sets.

In another paper, examine in what way inheritance metrics contribute to predicting fault proneness in software. They have used 65 datasets with C&K and inheritance metrics to assess the influence of inheritance on software fault prediction. They divided the dataset into a dataset with inheritance and C&K and C&K minus inheritance for assessment of outcomes. Algorithm ANN is applied. Recall, Accuracy, F1 measures, TNR, and Precision are computed for gaging performance. Assessment is constructed and outcomes illustrations a satisfactory influence of inheritance metrics in software fault prediction. The testing community may securely employment inheritance metrics in forecasting faults in software. Furthermore, elevation in inheritance is unsuitable since it hypothetically leading towards faults in software.

## Experimental methodology

The objective of this experimentation is to proves the effectiveness of inheritance metrics to handle unlabeled datasets in SFP applying unsupervised learning models and the secondary objective is to identify clusters as faulty or fault-free through the novel average mechanism. Literature review divulges that the metric suite of Chidamber & Kemerer (C&K) is extensively utilized in software fault prediction. Establishing this fact, we plan to evaluate the outcomes taken from C&K metrics with the results of Inheritance with C&K (Inheritance^+*C*&*K*^) to find the superior outcomes to see the effectiveness of inheritance metrics or otherwise.

In the aforesaid experiment, the C&K dataset comprises six metrics {wmc, cbo, rfc, lcom, dit, noc} and another dataset Inheritance^+*CK*^ comprises eight metrics, including C&K metrics {dit, wmc, noc, cbo, rfc, lcom} and inheritance metrics {ic, mfa}. The source datasets for this experiment consist of 10 data sets which including two Inheritance metrics and six C&K metrics. Afterward, the individual source dataset is separated into further two derived data sets specifically Inheritance^+*C*&*K*^ (8 metrics) and C&K (6 metrics) to assess for superior. Lastly, the outcomes of both the derived data sets are shown in Section “Results/Comparison Phase”.

**Figure 1:**
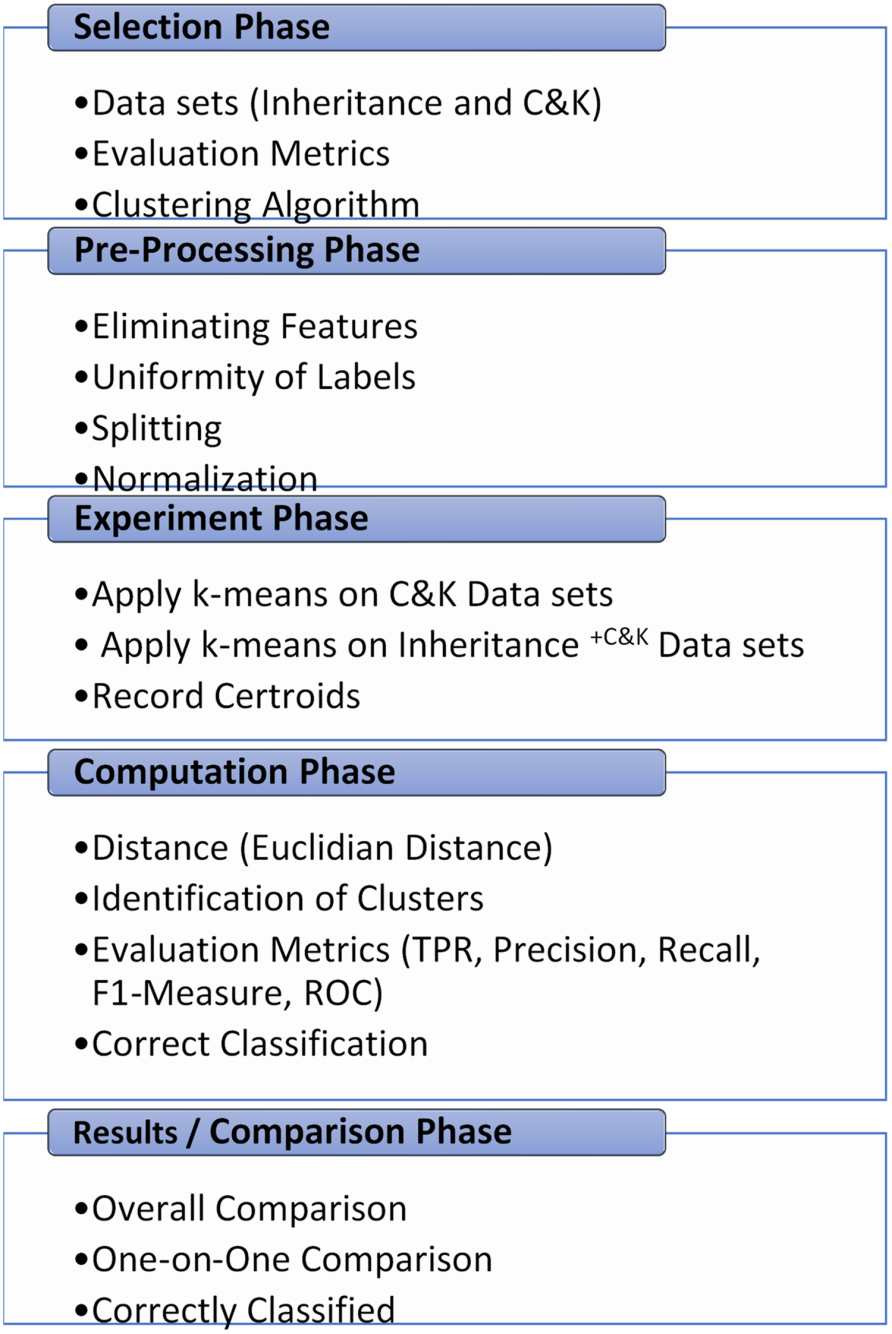
Experimental methodology.

The experimental methodology comprises five interconnected phases, as displayed in Fig. 1. The phases consist of selection phase, pre-processing phase, experiment phase, computation phase, and results/comparison phase. The first phase involves the careful selection of inheritance metrics, C&K metrics, clustering algorithm, and evaluation metrics for unsupervised learning. The source datasets selection is the basis of two conditions, shown in Fig. 1, it should be public datasets and must contain Inheritance and C&K metrics.

Subsequently, in the pre-processing phase, additional metrics other than C&K and inheritance are eliminated. Derived datasets C&K and Inheritance^+*C*&*K*^ are generated and make them harmonious. Then these generated datasets are utilized in the experiment phase where algorithm K-means clustering is performed on both the derived datasets and centroids of each cluster are recorded.

Next, in the computation phase distance is computed using the Euclidian formula, an evaluation matrix is calculated. Further TRP, Precision, Recall, F1-Measure, and ROC are calculated for both the generated datasets. Lastly, the average is taken to classify the faulty or fault-free clusters.

Lastly, in the final phase, several aspects are formulated to compare the results to ascertain the effectiveness of inheritance metrics to handle unlabeled datasets and marking clusters as faulty and fault-free basing on the average of centroids of metrics.

**Figure 2:**
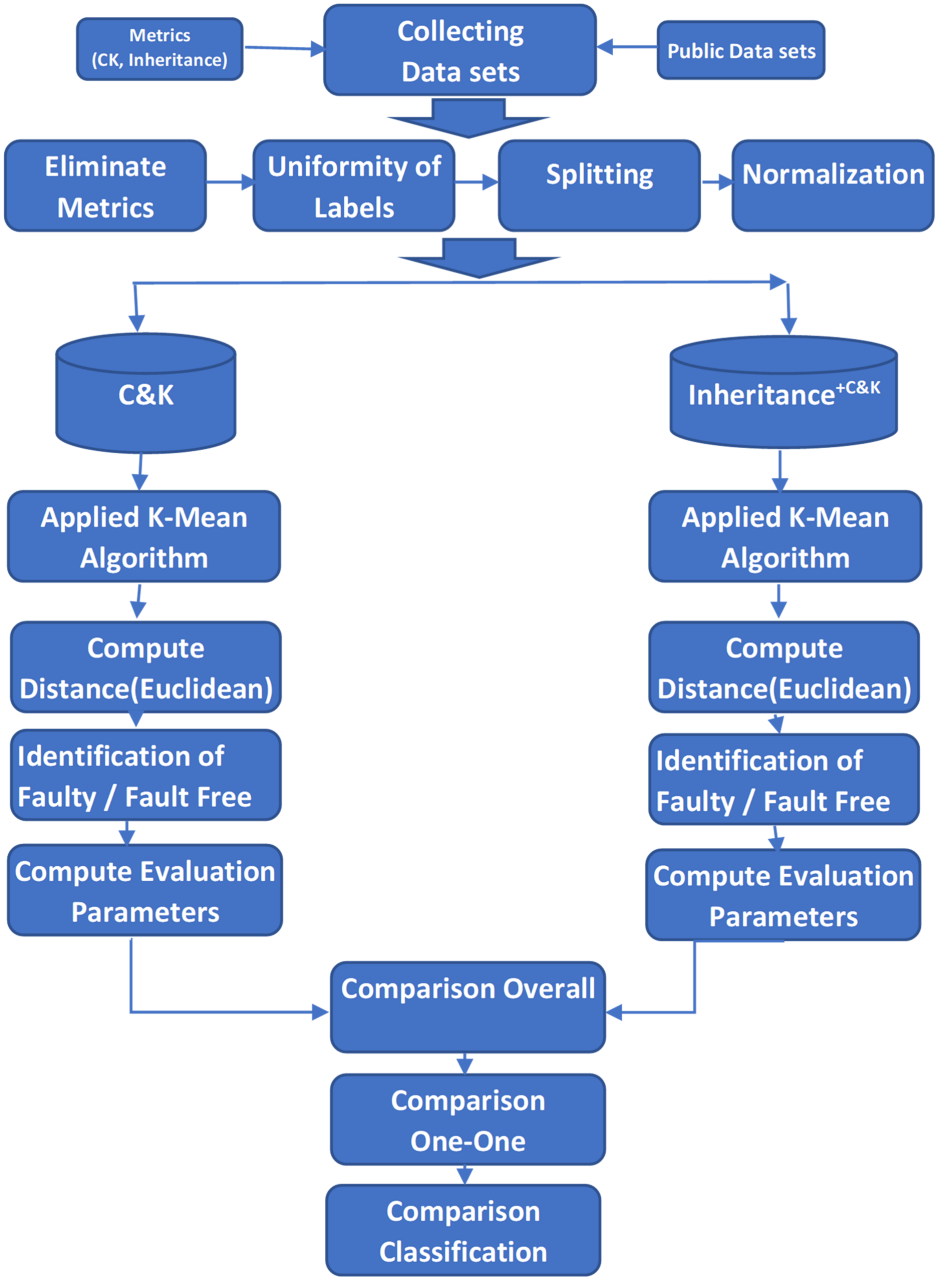
Experimental activity flow.

The activity diagram demonstrates the complete workflow and associated activities of the experiments in Fig. 2.

### Selection phase

This phase consists of picking and chooses of inheritance, C&K, and evaluation metrics along with finding a clustering algorithm. The selection of metrics is based on two conditions explained as follows:

1. **Inheritance and C&K Metrics Selection.** The elementary purpose of collection is to take a group of data that encompasses C&K and inheritance metrics, discussed briefly in section two. We merely pick out those metrics which abide by the following conditions.

2. **Public Dataset.** This condition is important to meet since the information of fault for software projects is exceptionally a lesser amount of reachable. The reason for this, the software fault data for enterprises or commercial projects are gathered in private software repositories whose availability is private. Alternatively, within a small software project’s fault data is insufficient. Consequently, marked data is infrequently accessible. The reachability of public data set will permit the assessment of the inheritance metrics in software fault prediction. Ultimately, 10 source data sets having C&K and inheritance metrics are discovered (Jureczko & Madeyski, 2010; Menzies & Di Stefano, 2004; Menzies et al., 2004; Softlab, 2019; Niu & Mahmoud, 2012; D’Ambros, Lanza & Robbes, 2010; Wagner, 2010; Abdelmoez, Goseva-Popstojanova & Ammar, 2006; Abdelmoez et al., 2005; Monarchi & Puhr, 1992; Shepperd et al., 2013). The two metrics of inheritance are the functional abstraction measure (mfa) and inheritance coupling (ic). The C&K metrics suite be made up of depth of the Inheritance Tree (dit), class-weighted method (wmc), number of children (noc), response for a class (rfc), coupling between objects (cbo), lack of cohesion in the methods (lcom).

All ten data sets are situated on tera-PROMISE (Boetticher, 2007) and D’Ambros, Lanza & Robbes (2010) repositories. Table 4 displays the details of these datasets wherein column one displays the label of the source data set and version if existed. The number of instances in column two, the percentage of faults in column three, and the sum of metrics are displayed in column four. Overall, six metrics of C&K and two inheritance metrics are in these source data sets, where ✓ is indicated in the case where metric is appearing in the related source data set.
Metrics are biasing towards correlation as these emphasize the associated features of Object Oriented here it is inheritance. A superior rate of correlation greater than equal to zero point seven or less than equal to minus zero point seven is a classification of duplication which must be excluded. In the study (Aziz, Khan & Nadeem, 2021) the Pearson r and the Spearman p correlation coefficient for the pairs discovered in public data sets are computed. The findings revealed that all combinations are positively correlated.

**Table 4:** Source datasets.

Dataset Name	# Ins	% Faulty	#F	wmc	lcom	cbo	rfc	dit	noc	ic	mfa
ant-1.7	745	22	8	✓	✓	✓	✓	✓	✓	✓	✓
e-learning	64	9	8	✓	✓	✓	✓	✓	✓	✓	✓
iny-1.1	111	57	8	✓	✓	✓	✓	✓	✓	✓	✓
nieruchomosci	27	37	8	✓	✓	✓	✓	✓	✓	✓	✓
poi-1.5	237	59	8	✓	✓	✓	✓	✓	✓	✓	✓
serapion	45	20	8	✓	✓	✓	✓	✓	✓	✓	✓
synapse-1.0	157	10	8	✓	✓	✓	✓	✓	✓	✓	✓
szybkafucha	25	56	8	✓	✓	✓	✓	✓	✓	✓	✓
tomcat	858	8	8	✓	✓	✓	✓	✓	✓	✓	✓
xalan-2.7	909	98	8	✓	✓	✓	✓	✓	✓	✓	✓

3. **Evaluation Metrics.** The classification models of machine learning are assessed using their accomplishment once categorizing the unidentified occurrences. The confusion matrix is a method to reveal the performance of the algorithm, Catal computed many metrics originated through the confusion matrix (Malhotra & Jain, 2012). Also, Malhotra et al. suggest a common explanation of numerous assessment measures utilized in software fault prediction. Conferring their judgments, TPR is the greatest frequently utilized assessment measure in software fault prediction, followed by Precision (Malhotra, 2015).

The Accuracy shows the percentage of the sum of correct forecasts among the sum of correct and incorrect predictions. Precision signifies the share of correctly classified faulty classes across the sum of classified faulty classes. The recall is a ratio of correctly predicted faulty classes in-between entire real classes which are faults prone. F-measure is to quantify the Accuracy of an experiment. It involves Precision and Recall both in the experiment. F1-score is the harmonic mean of Recall and Precision where F1-score achieving the finest value at 1 (ideal Recall and Precision) and the poorest at the value 0.

We selected TPR, Recall, Precision, F1-measure, and ROC to assess the model utilized within the paper.

4. **Clustering Algorithm.** K-means clustering is a form of unsupervised learning, that is used in the case of data without labels. The objective of this algorithm to find groups within data, where the number of groups denoted with variable k. Algorithm functions recursively to allocate every data instance to one of k clusters basis on the attributes which are imparted. Data marks are grouped basis on the attribute’s resemblance. The outcomes of the K-means algorithm are:

(a) Clusters k centroids, that may be used to put a tag on newly created data.
(b) The training data labels where all data points are allocated a distinct cluster.
In particular, by describing sets before looking at the data, clustering allows to find and analyze naturally constructed sets. Each centroid of a cluster is a collection of attribute values that will be generated by the resulting groups. The study of the centroid element weights can be used to qualitatively determine the type of group for which each cluster is suitable.

### Preprocessing phase

1. **Eliminating Metrics.** The source datasets comprise several metrics, for example, loc, ca, ce, I, and so on. Since our goal is to assess C&K and Inheritance metrics in the software fault prediction, thus all other metrics are dropped from the source datasets. This might affect the performance of fault prediction, nevertheless, it is to be expected to evaluate the effectiveness of inheritance metrics on software fault prediction.
2. **Labels Uniformity.** Every feature of source datasets have continuous values however discrepancy is located within the feature referred as BUG. That is solved with the followings rules.
(1) $$BUG = \left\{ \matrix{{\rm False}\;{\rm Defects = 0, N, No, FALSE} \cr {\rm True\;\; Otherwise}} \right\}$$
  Between this fault-free is described as FALSE and faulty is described as TRUE for all occurrences in the source data sets.
3. **Splitting.** Since our purpose is to measure the inheritance metrics effectiveness, so the source data set is split into two additional derived data sets namely Inheritance^+*C*&*K*^ and C&K. The derived dataset C&K comprises six metrics and the other derived data set Inheritance^+*C*&*K*^ comprises eight metrics. Conclusively, Table 5 is separated into two portions to display the figures of derived data sets which are created by splitting source datasets. The first column displays the data set name with the version if it exists. In succeeding columns six features of the first derived dataset C&K and eight features of derived dataset Inheritance^+*CK*^ where ✓ is indicated in the case where metric is appear in the associated datasets.
4. **Data Normalization.** Normalization is utilized to scale the attribute data to decreases in the range between minus one to one or zero to one. It is usually effective in classification algorithms. It is typically required when processing attributes on a varied scale, otherwise, it might lead to a reduction in the usefulness of a vital similarly crucial attribute (on a smaller scale) as other attributes holding values on the higher scale.
  Basically, in the situation of numerous attributes and these are having values on different scales, this might lead to poorer data models during employing data mining functions. Therefore, these are normalized to take each attribute on an equivalent scale. Techniques, for example, Min–Max, Z-Score, and Decimal scaling are being applied for the normalizing of data.
  In this experimentation, the Min–Max technique (Jain & Bhandare, 2011) is applied to normalize the data using the R function mmnorm. Min–Max normalization performs a linear conversion on the original data. Each of the actual data of attribute is mapped to a normalized value which lies in the range of 0 to 1. The Min–Max normalization is calculated by using the equation:
$Normalized(d) = (d)\prime = \displaystyle{{d - min(P)} \over {max(p) - min(p)}}$
  In the equation above, the min(p) and max(p) denote the minimum values and maximum values of the features, correspondingly.

**Table 5:** Grouped and final datasets for experiment.

Dataset Name	#F	C&K						Inheritance +C&K								
		wmc	lcom	cbo	rfc	dit	noc	#F	wmc	lcom	cbo	rfc	dit	noc	ic	mfa
ant-1.7	6	✓	✓	✓	✓	✓	✓	8	✓	✓	✓	✓	✓	✓	✓	✓
e-learning	6	✓	✓	✓	✓	✓	✓	8	✓	✓	✓	✓	✓	✓	✓	✓
iny-1.1	6	✓	✓	✓	✓	✓	✓	8	✓	✓	✓	✓	✓	✓	✓	✓
nieruchomosci	6	✓	✓	✓	✓	✓	✓	8	✓	✓	✓	✓	✓	✓	✓	✓
poi-1.5	6	✓	✓	✓	✓	✓	✓	8	✓	✓	✓	✓	✓	✓	✓	✓
serapion	6	✓	✓	✓	✓	✓	✓	8	✓	✓	✓	✓	✓	✓	✓	✓
synapse-1.0	6	✓	✓	✓	✓	✓	✓	8	✓	✓	✓	✓	✓	✓	✓	✓
szybkafucha	6	✓	✓	✓	✓	✓	✓	8	✓	✓	✓	✓	✓	✓	✓	✓
tomcat	6	✓	✓	✓	✓	✓	✓	8	✓	✓	✓	✓	✓	✓	✓	✓
xalan-2.7	6	✓	✓	✓	✓	✓	✓	8	✓	✓	✓	✓	✓	✓	✓	✓

### Experiment phase

The particulars concerning experimentation setup are enlightened in the followings lines:

DATASET: 10 derived data sets respectively for C&K and Inheritance^+*C*&*K*^, as displayed in Table 5.

TOOLS: Language R 3.4.3 (Rajnish & Bhattacherjee, 2005) in R Studio 1.1.383 (Rajnish & Bhattacherjee, 2006).

CLASSIFIERS: The utmost widespread method of unsupervised learning is the K-means algorithm for clustering. The key objective of this algorithm is to cluster alike data occurrences collectively and discover patterns in the said datasets. To accomplish this objective, this algorithm states the number of clusters denoted as (k) and after that collects the alike components into clusters. It begins by choosing the centroids, which is the initial point of the clusters. Subsequently, it allocates the occurrences to the neighboring centroids and afterward renews the locations of the centroids recursively till the centroids are stationary or the preset maximum times the iterations are reached.

A data set is given with (n) occurrences *S* = {*a*_1_,*a*_2_,…,*a*_*n*_} *in X*^*e*^, and an digit quantity (k), algorithm K-means intends to locate *M* = {*c*_1_,*c*_2_,…,*c*_*n*_}, the collections of centroids on the basis of the error function mentioned as under:

$E(C) = \sum\limits_{x \in {S^i} = 1,....k} min{\rm \parallel }x - {c_j}{{\rm \parallel }^2}$

As stated earlier, K-means allocates occurrences to each of the defined clusters as per resemblance among them. In order to determine the resemblance, it typically applies the Euclidean distance amongst the occurrence and the centroids. K-means is applied on all 10 derived datasets of C&K and Inheritance^+*C*&*K*^ respectively as shown in Table 5. K-means applied using ClusterR 1.2.1 package of R language. We use the “Elkan” variant of K-means. The reason for selection is that it uses the triangle inequality to efficiently map the data with well-defined clusters. However, the algorithms have relatively higher space complexity. Since we are interested in the binary classes of SFP, we initialize the value of k as 2. The initial centroid is determined randomly in 100 different initializations to avoid biases. Moreover, we performed 300 iterations with tolerance as low as 0.0001. Finally, the quality of clusters is measured in terms of the Silhouette Score. A clustering process is applied on both the derived datasets denoted as C&K and inheritance^+*C*&*K*^ as displayed in Table 5 to group its instances into clusters based on their similarity. We begin by grouping instances into two groups of clusters. We use the K-means algorithm as the most popular clustering technique. The centroids of all the metrics are recorded for both C&K and inheritance^+*C*&*K*^ datasets.

### Computation phase

After applying the K-means algorithm on 10 datasets each of C&K and Inheritance^+*C*&*K*^ further activities includes the computation of various elements of the experiment. Firstly, distance needs to be calculated through the Euclidean distance formula. Secondly, identification of cluster being faulty or fault-free and lastly computation of performance measure to draw findings out of this experiment. These activities are explained subsequently in the following lines as under:

1. **Computation of Distance.** The first activity to compute the distance of each instance to identify which instance fall in which cluster. Therefore distance of each instance is computed for C&K and Inheritance^+*C*&*K*^ datasets through the following Euclidean distance formula.
$d(p,q) = \sqrt {{{({q_1} - {p_1})}^2} + {{({q_2} - {p_2})}^2} + ... + {{({q_n} - {p_n})}^2}}$
  where *p*, *q* are two points in Euclidean n-space. *q*_*i*_, *p*_*i*_ are euclidean vectors, starting from the origin of the space(initial points) and *n* = n-space.
2. **Identification of Clusters.** The next step is to identify a cluster as faulty or faulty free. In this regard, the average is taken to all the metrics of a cluster. Resultantly, the larger value declared as the faulty and smaller value of average is considered as fault-free. The process follows the following formula:
(2) $$f({C_{1,2}}) = \left\{ \matrix{{\rm Faulty} \hfill \cr {\rm Max}\left({{\sum_{i = 1}^n value({M_i},{C_1})} \over {{\rm \parallel }M{\rm \parallel }}},{{\sum_{i = 1}^n value(Mi,{C_2})} \over {{\rm \parallel }M{\rm \parallel }}}\right) \cr {\hskip-5.6pc}{\rm Fault\ Free\ Otherwise} } \right \}$$
  where *C* referring cluster 1 and 2, *M* represent metrics, and ∥*M*∥ represent the cardinality of metrics in the formula.
  In the experiment, the C&K dataset has ∥*M*∥ of C&K dataset is six and Inheritance^+*C*&*K*^ has eight. Each dataset has two clusters. So, after applying the above-mentioned formula the Table 6 shows the results. The first column shows the name of the base dataset, the second column shows the predicted cluster as 0 fault-free and 1 as faulty. Column 3–8 the centroids values of respective metrics of the C&K dataset and column 9 shows the average for the first cluster in the first row and the average of the second cluster in the next row against each base dataset. Similarly, column 10–17 shows the centroids values and in column 18 average of clusters one and two in first and a subsequent row of the base dataset for Inheritance^+*C*&*K*^.
  Findings revealed that the Inheritance^+C&K^ has correctly classified the faulty and fault free clusters but C&K has not correctly classified clusters of serapion and synapse-1.0 datasets.
3. **Computation of Performance Measures.** All ten data sets displayed in Table 5 have been utilized for model construction and authenticating K-means clustering. Model construction of K-means is accomplished employing ClusterR 1.2.1 package of R 3.4.3 language. K-means is applied on both the datasets and TPR, Precision, Recall, F1-score, ROC, and percentage of corrected classified instances are computed for C&K and Inheritance^+*C*&*K*^ datasets. Accumulative findings are presented in Table 7.

**Table 6:** Identification of faulty or fault free clusters.

Datasets	Cluster	C&K							Inheritance +C&K								
		wmc	lcom	cbo	rfc	dit	noc	Average	wmc	dit	noc	cbo	rfc	lcom	mfa	ic	Average
ant-1.7	0	8.16	3.58	0.45	9.62	22.92	45.16	14.98	8.36	2.98	0.57	9.76	21.91	44.19	0.82	0.78	11.17
	1	9.08	1.23	0.59	11.41	21.16	108.61	25.35	9.21	0.99	0.52	11.90	21.52	131.58	0.02	0.02	21.97
e-learning	0	5.21	1.34	0.31	4.90	9.14	16.24	6.19	5.87	1.17	0.39	4.78	9.96	20.00	0.08	0.04	5.29
	1	7.74	4.51	0.94	4.91	15.46	31.51	10.85	7.00	4.15	0.80	4.98	14.07	27.17	0.86	0.71	7.47
iny-1.1	0	5.21	1.34	0.31	4.90	9.14	16.24	6.19	9.32	1.03	0.18	10.41	25.81	86.27	0.01	0.14	16.64
	1	7.74	4.51	0.94	4.91	15.46	31.51	10.85	13.81	3.25	0.53	10.47	48.63	89.09	0.72	0.94	20.93
nieruchomosci	0	8.00	1.56	0.00	5.89	9.11	8.00	5.43	4.94	1.00	0.00	4.12	16.71	4.41	0.00	0.00	3.90
	1	2.78	1.94	0.00	3.00	25.06	1.28	5.68	3.80	3.20	0.00	3.70	17.70	2.00	0.82	0.50	3.97
poi-1.5	0	9.10	1.00	1.90	11.19	23.89	26.79	12.31	10.37	1.00	1.86	11.89	28.38	60.52	0.00	0.04	14.26
	1	16.01	2.12	0.05	6.53	29.81	133.33	31.31	15.30	2.13	0.05	6.02	27.04	113.39	0.52	0.93	20.67
serapion	0	11.56	6.44	0.78	8.22	59.89	55.56	*23.74*	6.31	1.69	0.66	6.83	21.31	16.57	0.25	0.03	6.71
	1	5.42	1.75	0.64	7.00	20.00	8.81	*7.27*	7.80	6.20	0.70	8.70	51.30	23.70	0.97	0.70	12.51
synapse-1.0	0	22.67	1.52	0.00	23.90	73.33	224.86	*57.71*	6.27	2.60	0.31	12.58	27.64	12.00	0.63	0.91	7.87
	1	5.82	1.60	0.43	11.53	23.12	10.32	*8.80*	8.79	1.18	0.40	13.43	30.71	49.88	0.05	0.02	13.06
szybkafucha	0	7.41	1.00	0.00	6.00	11.76	22.71	8.15	7.41	1.00	0.00	6.00	11.76	32.71	0.00	0.00	7.36
	1	5.38	7.00	0.00	10.38	28.88	5.50	9.52	5.38	7.00	0.00	10.38	28.88	5.50	0.91	1.00	7.38
tomcat	0	10.69	2.71	0.35	6.44	33.46	80.28	22.32	9.73	2.76	0.36	6.32	31.45	65.73	0.77	0.71	14.73
	1	14.21	1.00	0.37	8.34	32.89	227.48	47.38	14.63	1.04	0.36	8.34	34.10	229.90	0.01	0.02	36.05
xalan-2.7	0	7.85	3.69	0.42	11.93	26.10	58.34	18.06	8.83	3.37	0.61	12.50	27.40	66.32	0.88	1.16	15.13
	1	14.50	1.28	0.62	12.51	33.70	207.23	44.97	14.96	1.06	0.37	11.74	33.92	240.41	0.01	0.02	37.81

**Table 7:** Evaluation parameters results.

Datasets	Inheritance + C&K					C&K				
	TP rate	Precision	Recall	F-measure	ROC area	TP rate	Precision	Recall	F-measure	ROC area
ant-1.7	0.622	0.774	0.622	0.69	0.477	0.526	0.83	0.526	0.644	0.565
e-learning	0.627	0.902	0.627	0.74	0.414	0.475	0.966	0.475	0.636	0.637
iny-1.1	0.771	0.468	0.771	0.583	0.552	0.854	0.466	0.854	0.603	0.554
nieruchomosci	0.941	0.727	0.941	0.821	0.671	0.882	0.652	0.882	0.75	0.541
poi-1.5	0.738	0.717	0.738	0.727	0.655	0.858	0.565	0.858	0.682	0.445
serapion	0.917	0.805	0.917	0.857	0.514	0.889	0.8	0.889	0.842	0.5
synapse-1.0	0.688	0.874	0.688	0.77	0.406	0.603	0.934	0.603	0.733	0.614
szybkafucha	0.989	0.647	0.976	0.786	0.786	0.909	0.625	0.909	0.741	0.74
tomcat	0.627	0.909	0.627	0.742	0.48	0.602	0.911	0.602	0.725	0.489
xalan-2.7	0.597	0.998	0.597	0.747	0.753	0.511	0.998	0.511	0.676	0.71
Sum	7.517	7.821	7.504	7.463	5.708	7.109	7.747	7.109	7.032	5.795
Median	0.713	0.790	0.713	0.745	0.533	0.729	0.815	0.729	0.704	0.560
Average	0.752	0.782	0.750	0.746	0.571	0.711	0.775	0.711	0.703	0.580
SD	0.140	0.145	0.138	0.071	0.130	0.172	0.176	0.172	0.066	0.091

### Results/comparison phase

The utmost significant objective of this paper is experimentation and to validate the effectiveness of inheritance metrics to classify unlabeled datasets employing unsupervised learning model K-means clustering, whereas the subsidiary purpose is to attain the best findings of the algorithms of machine learning. In addition, identification of clusters as faulty or fault-free is also a part of this paper. So that is why we have exactly filtered data sets and designed the experiments. Table 7 demonstrates the scores of the experiments in which TPR, Precision, Recall, F1-score, and ROC are computed for 10 data sets of C&K and Inheritance^+*C*&*K*^.

**Figure 3:**
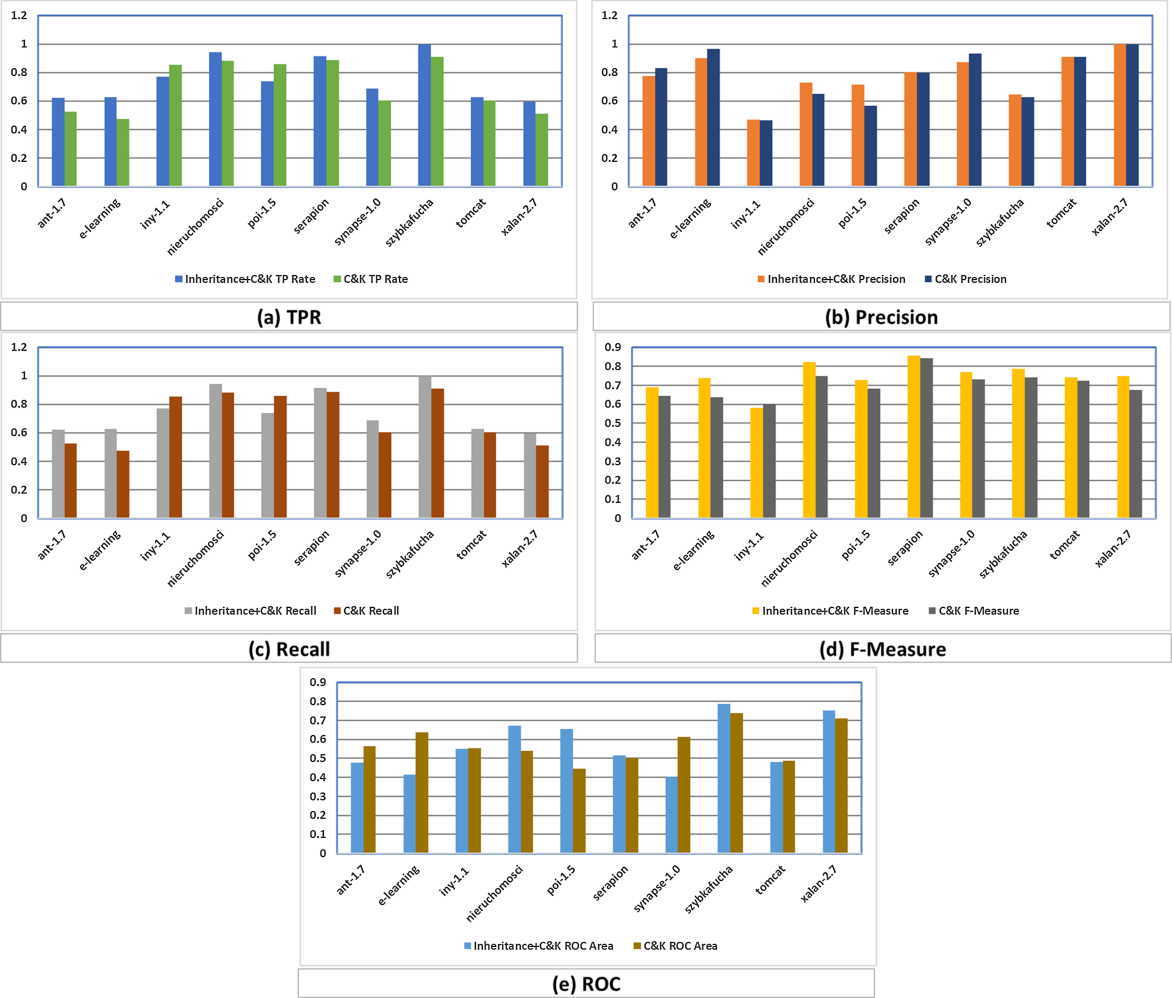
(A–E) Assessment among Inheritance^+*CK*^ and C&K.

In this regards, Fig. 3 graphically compares the performance differences of K-means in C&K and Inheritance^+*C*&*K*^ through evaluation parameters of TPR, Precision, Recall, F1-Measure and ROC which reveal that Inheritance^+*C*&*K*^ has superior results overall.

TPR assessment of Inheritance^+*CK*^ and C&K is shown in Fig. 3A where eight times Inheritance^+*CK*^ has better results while in two cases C&K is superior.

In case of Precision, Fig. 3B depicts that six times Inheritance^+*CK*^ has better results while in four cases C&K is superior.

In case of Recall, Fig. 3C depicts that eight times Inheritance^+*CK*^ has better results while in two cases C&K has superior results.

In case of F1-Measure, Fig. 3D depicts that nine times Inheritance^+*CK*^ has better results while in only one case C&K has superior result.

In case of ROC, Fig. 3E depicts that six times Inheritance^+*CK*^ has better results while in four cases C&K has superior results.

**Figure 4:**
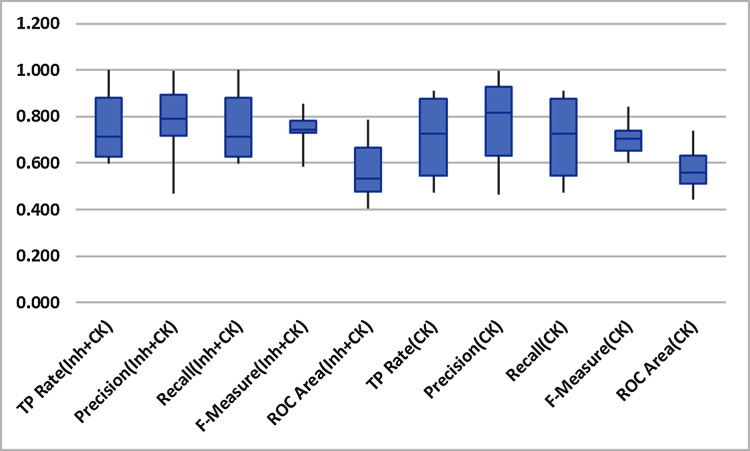
Box-plot.

Figure 4 indicates the non-existence of outliers in the findings throughout all the datasets. So it can thus be safely acknowledged that the means of performance methods are not prejudiced. Therefore revealed performance of Inheritance^+*CK*^ metrics predominates.

In order to do further validation of the effectiveness of inheritance in software fault prediction using unlabeled data, the following evaluations are carried out among both the data sets.

#### Overall comparison

In the perspective of endorsement, the overall evaluation among C&K and Inheritance^+*C*&*K*^ datasets to find out which have produced superior findings. In this respect, bottom four rows of Table 7 displayed the overall sum, median, average and standard deviation in respect of TPR, Precision, Recall, F1-score and ROC for both the derived data sets. Their results are described in the following lines:

1. Overall sum of dataset Inheritance^+*C*&*K*^ is 7.517, 7.821, 7.504, 7.563, 5.708 and C&K data set is 7.109, 7.747, 7.109, 7.032, 5.795 for TPR, Precision, Recall, F1-score, and ROC.
2. Overall median of data set Inheritance^+*C*&*K*^ is 0.713, 0.790, 0.713, 0.745, 0.533 and data set C&K is 0.729, 0.815, 0.729, 0.704, 0.560 for TPR, Precision, Recall, F1-score, and ROC.
3. Overall average of data set Inheritance^+*C*&*K*^ is 0.752, 0.782, 0.750, 0.746, 0.571, and data set C&K is 0.711, 0.775, 0.711, 0.703, 0.580 for TPR, Precision, Recall, F1-score, and ROC.
4. Overall standardization of data set Inheritance^+*C*&*K*^ is 0.140, 0.145, 0.138, 0.071, 0.130, and data set C&K is 0.172, 0.176, 0.172, 0.066, 0.091 for TPR, Precision, Recall, F1-score, and ROC.

The conclusion is drawn keeping in mind the above-mentioned arguments where Inheritance^+*C*&*K*^ possesses superior scores for factors sum and the average for all evaluation metrics. But the results of the median and the standard deviation are identical for both the datasets.

#### One-one comparison

Outcomes of the experimentation comprising TRR, Precision, Recall, F1-score, and ROC are displayed in Table 7 for Inheritance^+*C*&*K*^ and C&K data sets. So, to make line-wise assessment, the values of evaluation metrics of both the data sets are equate to determine the larger values. The findings are condensed in Table 8 which demonstrates that:

**Table 8:** One to one comparison.

Group	TP rate	Precision	Recall	F-measure	ROC area
Inheritance+C&K (#)	8	8	8	9	6
C&K (#)	2	3	2	1	4
Inheritance+C&K (%)	80	73	80	90	60
C&K (%)	20	27	20	10	40

1. The F1-measure shows that inheritance^+*C*&*K*^ has superior results in 9 datasets whereas C&K is superior in only one dataset. So Inheritance^+*CK*^ attained 90% of total datasets as compare to 10% for C&K.
2. TRR, Precision, and Recall showed inheritance^+*C*&*K*^ has superior results in 8 datasets whereas C&K is superior in 2 datasets. So inheritance^+*C*&*K*^ attained 80% of total datasets as compare to 20% for C&K.
3. Precision displayed that the inheritance^+*C*&*K*^ retains better scores in 8 data sets and C&K is better in 3 datasets. Therefore Inheritance^+*C*&*K*^ achieved 73% overall as making a comparison with C&K which achieved 27% overall.
4. The ROC indicates inheritance^+*C*&*K*^ possesses better scores in 6 data sets while C&K is better in 4 data sets. Thus Inheritance^+*C*&*K*^ achieved 60% of overall data sets as making a comparison with C&K which achieved 40%.

**Figure 5:**
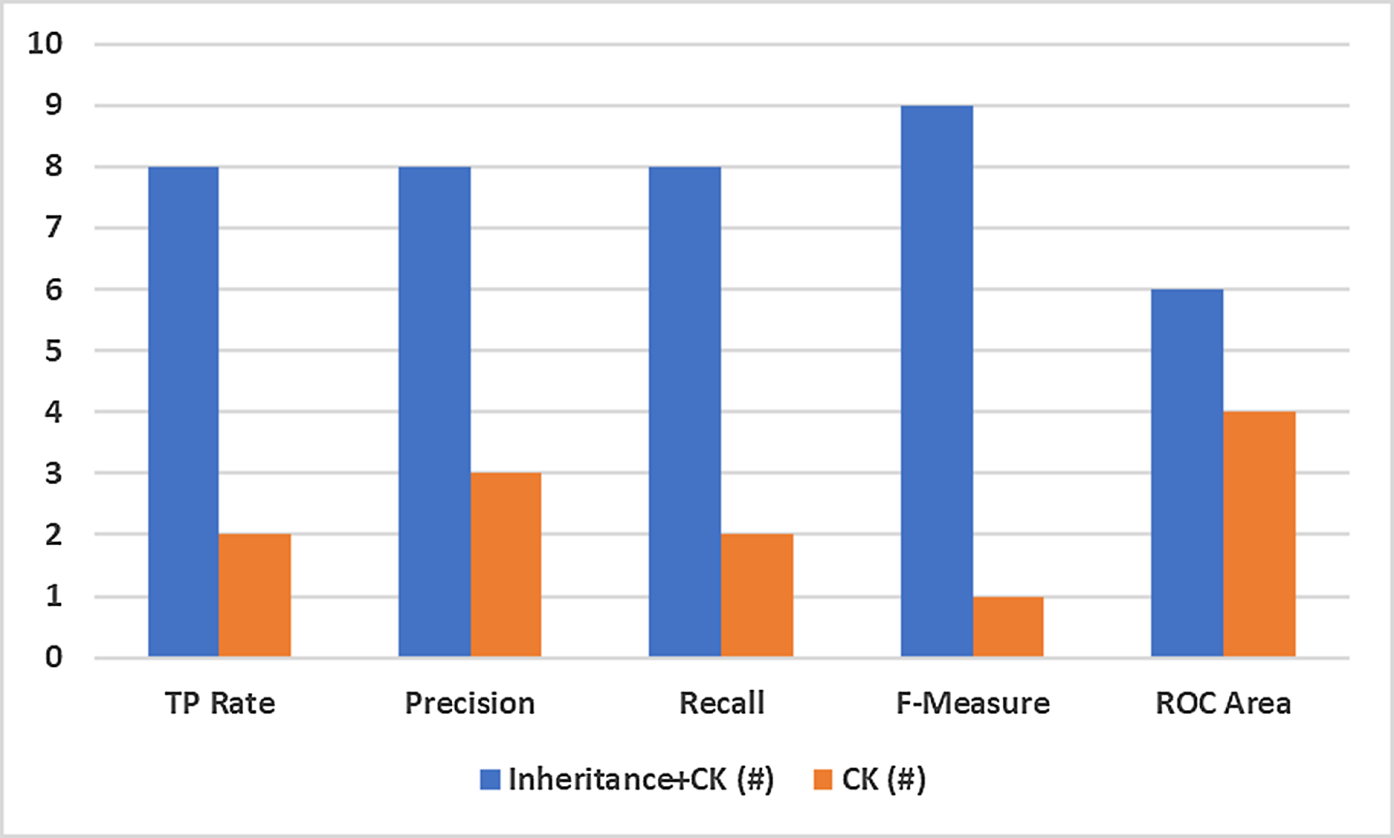
One to one comparison.

Basing on ten datasets, Inheritance^+*C*&*K*^ datasets have better scores to making the one-to-one comparison with C&K for TRR, Precision, Recall, F1-score, and ROC. The finds are depicted graphically in Fig. 5.

#### Comparison of correct classification

Mostly C&K metrics suit is regarded as superior performer in software fault prediction domain. Thus to determine inheritance metrics will be beneficial to correctly classified instances or otherwise. In this regards, K-means clustering is applied on 10 selected datasets which are split into Inheritance^+*C*&*K*^ and C&K datasets as displayed in Table 5 and score of evaluation metrics are depicted in Table 7. The findings are summarized in Table 9 which are:

**Table 9:** Comparison of correct classification.

Datasets	Inheritance^+C&K^	C&K
	Correctly classified (%)	Correctly classified (%)
ant-1.7	56	54
e-learning	59.38	50.00
iny-1.1	52.25	51.35
nieruchomosci	74.07	62.96
poi-1.5	67.09	52.32
serapion	75.56	73.33
synapse-1.0	63.06	60.51
szybkafucha	76	72
tomcat	60.13	58.25
xalan-2.7	60.13	51.63

1. All percentage depicted that Inheritance^+*C*&*K*^ datasets have superior results in all 10 datasets as compare to C&K datasets.
2. Min and Max percentage of the correctly classified instance are 52–76% by Inheritance^+*C*&*K*^ whereas 50–73% by C&K dataset in all 10 base datasets.
3. The difference between Inheritance^+*C*&*K*^ and C&K is 2% to 15% respectively in all 10 base datasets.

**Figure 6:**
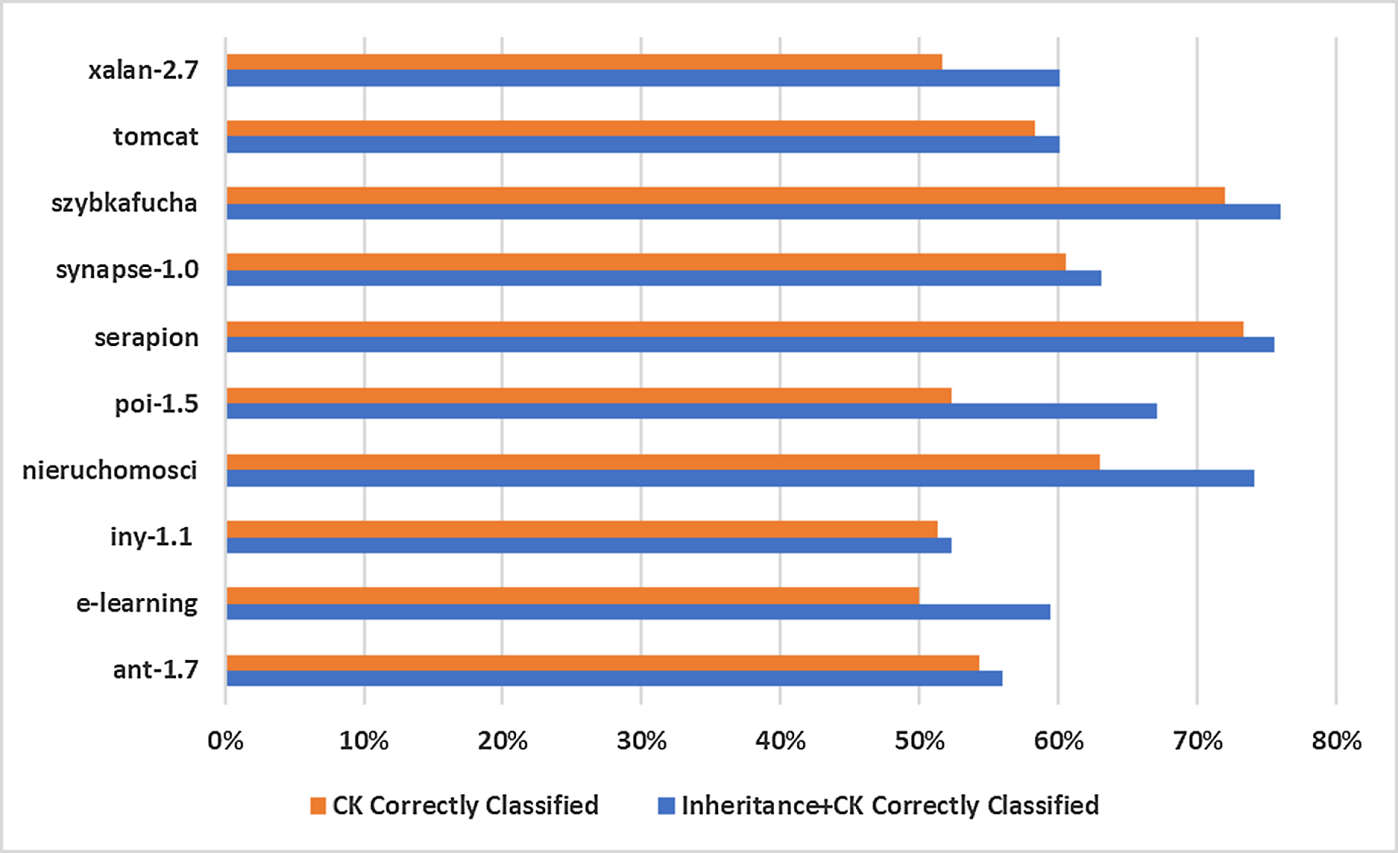
Graphical representation of correct classification.

Basing on to all 10 datasets, Inheritance^+*C*&*K*^ datasets achieved significantly better than C&K dataset to correctly classified instances. The results are displayed graphically in Fig. 6.

## Threats to validity

This research influenced the datasets obtained from the repositories of tera-PROMISE and NASA wherein there is not sufficient evidence presented regarding the faults whether these are appropriate to some specific kind of faults in software.

In the paper, a fault is not indicated for some specific type of fault for software. Consequently, the prediction might not be widespread to all types of faults associated with the software. Likewise, the chosen datasets encompass the small number of software products that are diverging in team, design, and scope. The manifestation of fault might not be the effect of inheritance only.

Lastly chosen metrics of inheritance are not report entire aspects coupled by inheritance in software. Thus the generality of these chosen metrics of inheritance might not be the outcome of entire facets of inheritance.

## Conclusion and future work

The paper portrayed the effectiveness of inheritance metrics to manage unlabeled datasets and an average-based mechanism to label a cluster as faulty or fault-free is validated in the background of SFP. The measurement is achieved via the experimentations on 10 openly accessible datasets using K-means clustering algorithms using unlabeled datasets. The projecting aptitude of the C&K metrics suit is recognized in the testing community, which predominates in our experimentations as well. Nevertheless increasing additional inheritance metrics to the C&K suite considerably improves the predictive ability specifically in the unlabeled datasets. Additionally applying an average base mechanism to mark a cluster as faulty and fault-free is easy and productive to overcome the label of clustering issue. This supports the effectiveness of the inheritance metrics in software fault prediction specifically in unsupervised learning.

The findings of this paper suggest that practitioners of the testing community be able to safely utilize metrics of inheritance in unsupervised learning to predict faults in software. Furthermore, the increase in the numbers in inheritance metrics suggests more chances of faults in the software. This instructs the developers/designers of software to maintain the inheritance aspect as low as possible.

As regards future work, we assume some researchers would rebuild our experimentation and try to assess inheritance metrics others the ones we have employed. Excitingly, machine learning techniques basing on regression for the prediction of faults utilizing inheritance metrics would be a remarkable job to be performed.

## Supplemental Information

10.7717/peerj-cs.722/supp-1 Supplemental Information 1 Dataset containing 64 tuples with fault percentage is 22%. The dataset contains eight software metrics.

10.7717/peerj-cs.722/supp-2 Supplemental Information 2 Dataset containing 111 tuples with fault percentage is 57%. The dataset contains eight software metrics.

10.7717/peerj-cs.722/supp-3 Supplemental Information 3 Dataset containing 237 tuples with fault percentage is 59%. The dataset contains eight software metrics.

10.7717/peerj-cs.722/supp-4 Supplemental Information 4 Dataset containing 45 tuples with fault percentage is 20%. The dataset contains eight software metrics.

10.7717/peerj-cs.722/supp-5 Supplemental Information 5 Dataset containing 25 tuples with fault percentage is 56%. The dataset contains eight software metrics.

10.7717/peerj-cs.722/supp-6 Supplemental Information 6 Dataset containing 157 tuples with fault percentage is 10%. The dataset contains eight software metrics.

10.7717/peerj-cs.722/supp-7 Supplemental Information 7 Dataset containing 858 tuples with fault percentage is 8%. The dataset contains eight software metrics.

10.7717/peerj-cs.722/supp-8 Supplemental Information 8 Dataset containing 909 tuples with fault percentage is 58%. The dataset contains eight software metrics.

10.7717/peerj-cs.722/supp-9 Supplemental Information 9 Calculation Sheet.

10.7717/peerj-cs.722/supp-10 Supplemental Information 10 Raw datasets.

10.7717/peerj-cs.722/supp-11 Supplemental Information 11 Script file to pre-process data sets using R platform.

10.7717/peerj-cs.722/supp-12 Supplemental Information 12 R platform scrip file to draw various graphs.

10.7717/peerj-cs.722/supp-13 Supplemental Information 13 R platform script file to apply k-mean clustering algorithm onto pre-process datasets.

## Additional Information and Declarations

### Data Availability

The following information was supplied regarding data availability: The raw datasets are available in the Supplemental File dataset.rar. The dataset contains C&K metrics with inheritance metrices. K-means is applied using R plate from and the final calculation is done in Excel.
